# Possible implications of animal models for the assessment of visceral pain

**DOI:** 10.1002/ame2.12130

**Published:** 2020-08-10

**Authors:** Bharata Regmi, Manoj K. Shah

**Affiliations:** ^1^ Department of Surgery and Pharmacology Agriculture and Forestry University (AFU) Rampur Chitwan Nepal

**Keywords:** animal model, inflammation, noxious stimuli, traction, visceral pain

## Abstract

Acute pain, provoked generally after the activation of peripheral nociceptors, is an adaptive sensory function that alerts the individual to avoid noxious stimuli. However, uncontrolled acute pain has a maladaptive role in sensory activity leading to development of a chronic pain state which persists even after the damage is resolved, or in some cases, in the absence of an initial local acute injury. Huge numbers of people suffer from visceral pain at least once during their life span, leading to substantial health care costs. Although studies reporting on the mechanism of visceral pain are accumulating, it is still not precisely understood. Therefore, this review aims to elucidate the mechanism of visceral pain through an evaluation of different animal models and their application to develop novel therapeutic approaches for treating visceral pain. To assess the nociceptive responses in viscera, several visceral pain models such as inflammatory, traction, stress and genetic models utilizing different methods of measurement have been devised. Among them, the inflammatory and traction models are widely used for studying the visceral pain mechanism of different disease conditions and post‐operative surgery in humans and animals. A hapten, 2,4,6‐trinitrobenzene sulfonic acid (TNBS), has been extensively used as an inflammatory agent to induce visceral pain. The traction model seems to cause a strong pain stimulation and autonomic reaction and could thus be the most appropriate model for studying the underlying visceral pain mechanism and for probing the therapeutic efficacies of various anesthetic and analgesics for the treatment of visceral pain and hyperalgesia.

## Graphical abstract

A large number of people experience visceral pain in their life span. Its mechanism has been explored utilizing inflammatory, traction, stress, and genetic animal models. Among these, the inflammatory and traction models are proved appropriate for studying the precise mechanism of visceral pain and to develop novel therapeutic approaches for treating it.

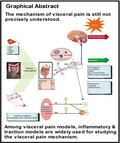

## 1 INTRODUCTION

Visceral pain is the most common but challenging problem seen in clinical patients. Approximately a quarter to a half of the world population experience visceral pain at least once during their life span, which leads to substantial health care costs.^1^ Inflammation, ischemia, or mesenteric stretching or distention of the hollow organs of the thoracic, pelvic, or abdominal cavities stimulate the afferent nociceptors to activate the pain pathways.^2^ The activated fibers transmit the nociceptive signals to the spinal dorsal horn (SDH) and then to the central nervous system (CNS), resulting in visceral pain.^3^ Visceral pain is diffuse in nature and is thereby very difficult to localize.^4^, ^5^

Animal models have played a pivotal role in our understanding of the mechanisms underlying the pathophysiology of visceral pain.^2^ Over the years, a number of studies have investigated the visceral pain provoked by traction of visceral organs in various species. Boscan et al^6^ investigated ovarian stimulation pain during ovariohysterectomy in bitches. Janyaro et al^7^ studied the visceral pain provoked by traction of ileocecal ligaments in an inflammatory ileal model of goats. Jones et al^8^ described the association of short term sensitization of mechanoreceptors with long term hypersensitivity in response to colonic distension in mice. DeBerry et al^9^ studied abdominal electromyographic (EMG) activity in response to noxious urinary bladder distention (UBD) in zymosan‐induced urinary bladder inflammation and outlined the effect of endogenous opioids in female rats. Similarly, Shah et al^10^ developed the 2,4,6‐trinitrobenzene sulfonic acid (TNBS)‐induced ileitis model in rats to study the pathophysiological mechanism of ileal Crohn's disease (CD). Wan et al^11^ used an ileitis‐provoked visceral hypersensitivity (VH) model to confirm the effects of electroacupuncture on VH through Janus kinase 2 (JAK2)/signal transducers and activators of the transcription 3 (STAT3) signaling pathway in the periaqueductal gray‐rostral ventromedial medulla‐SDH axis. Very recently, Shah et al ^12^ used TNBS‐induced ileitis goats to study VH and reported that electroacupuncture attenuates VH through down‐regulation of spinal PAR‐2 and CGRP. The persistent pancreatitis pain induced by injection of dibutyltin dichloride in the tail vein, along with 10% alcohol in drinking water, has also been investigated in rats.^13^ A number of studies have elucidated the association of stress with a lowered pain sensation thresholds and enhanced pain perception that parallels the altered brain circuit activity.^14^ During stress, a neuroendocrine hormone, ie a corticosteroid, is secreted in response to activation of the hypothalamic‐pituitary‐adrenal axis that not only acts at mineralocorticoid and glucocorticoid receptors throughout the body, but also promotes enhanced sensitivity of neurons to both noxious and innocuous stimuli, which in turn promotes the development of chronic pain.^15^ Zheng et al^16^ reported that chronic stress induces visceral as well as somatosensory hyperalgesia in a chronic, intermittent stress rat model. The epigenetic modulation of gene expression could be responsible for the mechanism underlying the persistent effects of stress on visceral sensitivity. A growing line of evidence shows that changes in DNA‐methylation and histone‐acetylation patterns within the brain and spinal cord of rats result in alterations in nociceptive signaling via increased expression of pro‐ and anti‐nociceptive gene expression.^17^, ^18^

Among the various animal models for studying visceral pain, the traction model seems most effective for studying the underlying mechanism of visceral pain and drug efficacy because it results in direct activation of the nociceptors and the signal that consequently releases histamine from the mesenteric mast cells is also stronger.^6^, ^19^, ^20^ The abdomen is the site most prone to both acute and chronic painful syndromes resulting from visceral diseases, referred pain coming from adjacent structures, and/or systemic injuries.^21^ The various diseases or disorders of abdominal and uro‐genital organs that result in visceral pain include obstruction, intussusception, neoplasms, volvulus, extra‐luminal compression, intra‐abdominal abscess, celiac disease, typhlitis, intestinal or biliary colic, gall stones, incarcerated or strangulated hernia, intestinal and mesentery infarcts, ovarian cysts, tumors, endometriosis and urolithiasis. Besides these, surgeries of the gastrointestinal tract and pelvic organs also produce visceral pain in animals and human beings.

This review of visceral pain research outlines the mechanism of visceral pain provoked by inflammation, stress, genetic and repetitive stimulation of visceral organs in various animal models.

## 2 NATURE OF VISCERAL PAIN

Visceral pain is the complex sensory experience arising from the viscera of the abdominal, thoracic, and pelvic cavities. We experience visceral pain because damaged or injured internal organs and tissues activate pain receptors. The pain may be accompanied by symptoms such as nausea, vomiting, changes in vital signs and emotional manifestations. There is no clear demarcation between the acute and chronic states of visceral pain.

Acute pain has an adaptive sensory function as it alerts the individual to avoid noxious stimuli,^20^ but uncontrolled acute pain has a maladaptive role in the sensory activity leading to development of a chronic pain state. In general, acute pain persists for a short time after injuries or inflammation. However, Kruszka and Kruszka^22^ reported that acute pelvic pain originating from the lower abdomen or pelvis may persist, alarmingly, for three months. Different pathological conditions such as ectopic pregnancy, hematoma of corpus luteum, ovarian cyst, ovarian tumor, ovarian torsion, acute salpingo‐oophoritis, fibroids, uterine cancer, cervical cancer, ovulation, appendicitis, fracture of pelvis and hernia provoke an intense acute pelvic pain which is challenging to investigate because the many symptoms are subtle and non‐specific. Some of these conditions may be life‐threatening (ectopic pregnancy, appendicitis and ruptured ovarian cysts) or may threaten fertility (pelvic inflammatory diseases, ovarian torsion).^22^ On the other hand, chronic visceral pain persists much longer, sometimes over a lifetime.^22^, ^23^, ^24^, ^25^ It is a symptom of several diseases and disorders but not, in and of itself, a disease process.

Visceral nociceptors do not have specialized receptors and neurotransmitters. Moreover, the neuroanatomy of visceral nociception – the neurotransmitters, receptors and ion channels that modulate visceral pain – is qualitatively and quantitatively different from that of the systems modulating somatic and neuropathic pain.^1^ Therefore, various animal models have been devised to study the mechanism underlying diseases causing visceral pain. It has been supposed that pain mechanisms serve as a natural protective mechanism against noxious stimuli by changing the physiology and behavior to reduce or avoid further damage, and promote recovery.^20^ A better understanding of the mechanism of peripheral and central sensitization can help to explain why prevention of sensitization is critical to recovery and will illuminate the multiple stages in the process that could be altered by different analgesic therapies. Moreover, the effective management of pain after surgery is a major welfare issue in human and animal practice. Management of acute postoperative pain remains suboptimal because nearly 80% of patients report subjectively moderate to extreme pain following surgery.^26^ Despite the various studies aimed at unraveling the mechanism of visceral pain, it is still less well understood than that of somatic pain. The reason may primarily be the diverse nature of visceral pain, compounded by multiple factors such as sexual dimorphism, psychological stress, genetic traits, and the nature of predisposing disease. These multiple contributing factors create a huge challenge for the development of an ideal animal model that closely mimics the disease condition being investigated. Over the past few decades, substantial numbers of animal models of visceral pain have been generated to improve management of pain and our understanding of the underlying mechanisms of visceral pain.

## 3 MECHANISM OF VISCERAL PAIN

**FIGURE 1:**
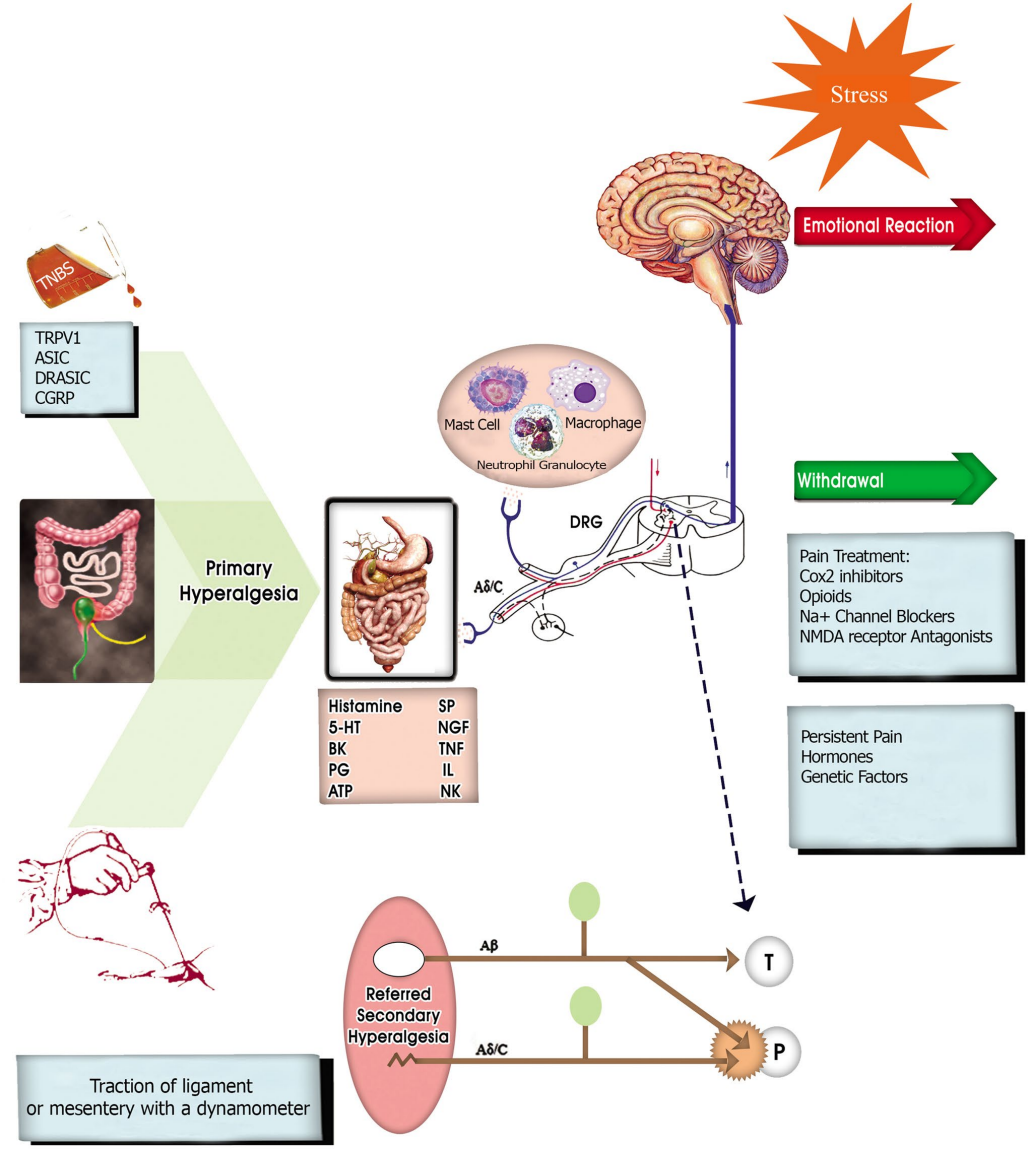
Diagram showing the basic mechanism of visceral pain and hyperalgesia. 5‐HT, 5‐hydroxytryptamine; ASIC, acid‐sensing ion channel; ATP, adenosine triphosphate; BK, bradykinin; CGRP, calcitonin gene related peptide; DRASIC, dorsal root acid sensing ionic channel; DRG, dorsal root ganglion; NGF, nerve cycloxygenase; NMDA, N‐methyl‐D‐aspartate; P, pain neurons; PG, prostaglandin; SP, substance‐P; T, tactile; TRPV1, transient receptor potential vanilloid type 1

Noxious stimuli including inflammatory agents and mechanical stimuli activate the nociceptors connected to unmyelinated C‐fibers and thinly myelinated Aδ‐fibers via specific receptors or ion channels sensitive to mechanical stimuli or inflammatory agents.^27^ Damaged tissue and inflammatory cells release different chemical mediators that activate or modify the stimulus response properties of nociceptors. The underlying mechanism of visceral pain and hyperalgesia is illustrated in Figure 1. Primary hyperalgesia, which occurs at the site of injury, is the consequence of increased input from nociceptors sensitized by the stimulus. These stimulated nociceptors connected to Aδ‐ and C‐afferent fibers transmit the amplified sensory discharges to the CNS, which results in an increase in the pain originating from the primary hyperalgesic area, called secondary hyperalgesia. Pain pathways (P) become activated in the CNS which stimulates tactile receptors connected to Aβ‐afferents leading to the activation of tactile pathways (T).^28^ The consequence of amplification of the nociceptive input from the injured area is access to pain neurons (P) resulting into touch‐evoked pain called allodynia.^29^ Secondary hyperalgesia is an increased sensitivity to pain occurring in areas adjacent to or even remote from the site of injury. It is the result of an alteration in the central processing of impulses from low threshold mechanoreceptors leading to activation of nociceptive neurons, thus evoking secondary hyperalgesia. This alteration is initially triggered and later maintained by the enhanced afferent discharges from the primary hyperalgesic area.^30^ In the case of referred visceral hyperalgesia, the primary focus is located in an internal organ, where nociceptors are sensitized by the originating stimulus and send enhanced discharges to the CNS. Somatic and visceral hyperalgesia are similar in the sense that both include a peripheral component of enhanced activity from nociceptors and a central component of alteration in the central processing of low threshold inputs, but the peripheral component can generate a secondary hyperalgesia in case of visceral hyperalgesia.^31^ Functional pain states triggered by various factors (genetic, hormonal etc), resulting in central alteration of the sensory processing that mediates referred secondary hyperalgesia, can be activated even in the absence of a peripheral stimulation of the nociceptors.^31^, ^32^

### 3.1 Peripheral sensitization

The physiology of pain in animals and humans involves the peripheral process of detecting a noxious stimulus (mechanical, thermal or chemical) and transmission of the impulses to the spinal cord. They are modulated and projected to the brain for central processing of the information, which determines the perception of the noxious stimulus.^28^ Except for nociceptive pain, all other types of pain are considered as clinical or pathological, often involving tissue damage with inflammation or nerve damage. The Aδ‐ and C‐fibers are the two types of sensory fibers conducting most of the nociceptive signals to the SDH, while the large Aβ‐fibers transmit other sensory information (pressure, touch, and vibration) to the CNS. They do not transmit pain impulses, but work in the spinal cord to modulate painful stimuli.^28^, ^29^ Aδ‐fibers are associated with intense and pricking pain, and rapidly conduct impulses (5‐20 m/s) while C‐fibers are associated with dull, burning pain and slowly conduct impulses (0.5‐1 m/s).^20^ Both types of fibers innervate skin and deep somatic/visceral structures but differ in their ratios. The reported ratio of Aδ‐ to C‐fibers is 1:1‐2 in cutaneous nerves, and 1:8‐10 in visceral nerves. Peripheral sensitization occurs when inflammation at the site of injury creates an increased response to a normally painful stimulus. These sensitized nociceptors evoke a stronger response to any given stimulus than in the normal state and their thresholds may be reduced such that even innocuous stimuli can activate them.^20^ Additionally silent nociceptors, not activated in the normal state, act as noxious stimuli to cause hyperalgesia and allodynia. In addition to sensitization at the peripheral tissue and pain receptors, the CNS is also sensitized.

### 3.2 Central sensitization

Animal studies involving direct electrophysiological recordings from spinal neurons suggest that central sensitization may also be an important mechanism in generating VH and pain. Central axons of first‐order neurons synapse onto second‐order neurons in the SDH. They terminate predominantly in laminae‐I, II, and V of the SDH on projection neurons and local interneurons.^33^ Laminae‐I and ‐II receive direct primary afferent inputs from Aδ‐ and C‐fibers.^28^ Modulation takes place mainly in the dorsal horn of the spinal cord where the first‐order neurons synapse with second‐order neurons.^34^ Traumatic injury and inflammation can also sensitize neurokinin transmission in the spinal cord to produce central sensitization. The prolonged firing of primary nociceptive afferents causes the increased release of glutamate neurotransmitter as well as the release of SP and brain‐derived neurotrophic factor onto the second‐order neurons. Recently it has been recognized that pro‐inflammatory mediators, such as cytokines and chemokines, released due to injury and peripheral inflammation^35^, ^36^ activate the glial cells of the spinal cord.^20^ These products increase neuronal excitability by activating receptors directly, up‐regulating the actions of excitatory amino acid receptors, or down‐regulating the actions of inhibitory receptors, such as GABA receptors.^37^ Nociceptive input can be amplified or attenuated in the SDH by a number of neuropeptides (SP, neurokinin glutamate, γ‐aminobutyric acid) released from neighboring neurons and descending pathways.^38^ For example, the wide dynamic range (WDR) neurons concentrated in deeper laminae (III‐V) respond to both noxious and non‐noxious stimuli which are transmitted by the Aδ‐, C‐ and Aβ‐fibers.^28^, ^34^, ^39^ Consequently, WDR neurons can play a role in the segmental suppression of pain.^40^ Primary (first‐order) neurons project from here to second‐order neurons in supraspinal centers in the ascending pathway of the dorsal horn of spinal cord which convey stimuli transduced by nociceptors to the brain‐stem, thalamus, limbic structure, and finally to neo‐cortex.^34^

## 4 MEASUREMENT OF VISCERAL PAIN IN LABORATORY ANIMALS

Quantitative measurement of pain severity, especially in non‐verbal animals that are used as disease models, can be difficult to obtain, but several developments in behavioral neuroscience are making the measurement of pain more consistent, automated and accurate.^41^ Some of the objective methods of visceral pain measurement used in different visceral pain models and experimental conditions in rodents have been summarized in Table 1.

**TABLE 1:** Different methods used to quantify visceral pain in laboratory animals

Animal	Animal model	Method	Purpose of the study	Findings	References
Mice	Experimental study	Telemetry	To study post laparotomy pain in laboratory mice by telemetric recording of heart rate and heart rate variability	Real‐time telemetric recordings of heart rate and heart rate variability found to be indicative of mild to moderate post laparotomy pain which cannot easily be detected by direct observation	Arras et al^57^
Rat	Inflammatory	Intracolonic manometry	To test the efficacy of pregabalin on visceral pain responses and colonic compliance	Pregabalin reduced the viscerosomatic and autonomic responses associated with CRD induced visceral pain and increased colonic compliance	Ravnefjord et al^62^
Mice	Inflammatory	Automated behavior analysis	To automate analysis of abdominal licking behavior associated with intraclonic capsaicin induced visceral pain	The neurokinin‐1 receptor antagonist GR205171A dose dependently inhibited capsaicin induced licking which was automatically detected by applying commercially available image analysis software	Hayashi et al^54^
Mice	Inflammatory	Burrowing	To investigate whether a change in burrowing behavior is a sensitive measure of animal welfare in murine models of colitis	Changes of spontaneous burrowing behavior correlate with the onset of inflammation in acute DDS induced colitis	Jirkof et al^53^
Rat	Inflammatory	Electrophysiology & behavioral studies	To study the reactive oxygen species mediated visceral pain related amygdala plasticity and behaviors	ROS contribute to visceral pain related hyperactivity of amygdala neurons and amygdala dependent behaviors through a mechanism that involves increased excitatory transmission and excitability of CeA neurons	Ji et al^73^
Rat	Stress	EMG	To study MS induced visceral hypersensitivity	Visceral hypersensitivity of MS rats is more pronounced in the post‐weaning period and slightly restored in adults. Thus, visceral hypersensitivity in the post‐weaning period might play a more meaningful pathophysiologic role in the formation of adult irritable bowel syndrome	Li et al^63^
Rat	Inflammatory	RGS, burrowing, CBS	To assess whether the RGS, CBS and burrowing could identify pain in an acute and chronic colitis model	RGS increased as DAI scores increased during both acute and chronic phases. Burrowing only decreased during the acute phase but CBS scores did not increase significantly during either colitis phase	Leung et al^49^

Abbreviations: CBS, Composite Behavior Score; CeA, Central Amygdala; CRD, Colorectal Distension; DAI, Disease Activity Index (assessing fecal blood, stool consistency and weight loss); DDS, dextran sulfate sodium; EMG, electromyography; MS, maternal separation; RGS, rat grimace scale.

### 4.1 Dynamic weight bearing

Dynamic weight bearing (DWB) is a non‐subjective and non‐reflexive method for the evaluation of inflammatory‐driven abdominal pain in a mouse model.^42^ Some researchers have used the DWB test as a tool for measuring non‐evoked inflammatory hyperalgesia in a mouse model,^43^ and it has also been shown to be an effective, objective and predictable test for studying both the pathophysiological mechanisms involved in arthritic nociception in mice and for evaluating novel analgesic drugs against arthritis.^44^

### 4.2 Grimace scale and burrowing

The grimace scale, originally developed to assess the pain in non‐speaking humans, was recently validated for pain assessment in several laboratory, farm and exotic animals (mouse, rat, rabbit, horse, cat, cattle, sheep, pig, ferret and seal).^45^ The rat grimace scale (RGS) rates pain from 0 (no pain) to 2 (severe pain) based on changes in four facial expressions, ie orbital tightening, nose/cheek flattening, ear changes and whisker changes.^46^ Langford et al^47^ did not observe grimacing in acute (<10 minutes) and chronic (>1 day) contexts, but subsequent researchers have reported seeing changes in facial expression lasting only a few seconds to minutes or extending to several months post injury.^45^ The feline grimace scale, however, includes five action units – ear position, orbital tightening, muzzle tension, whisker change and head position. The scores using this scale were reported higher during naturally occurring acute pain than in control cats.^48^

A recent study in an acute and chronic colitis model of rat showed that RGS increased during both acute and chronic phases but burrowing only decreased during the acute phase and suggested using RGS as an pain scale and welfare improvement tool.^49^ Rater training is mandatory for performing grimace scale research. At a minimum, observers need some years of species‐specific working experience to score the pain efficiently using the grimace scale. Burrowing, a spontaneous and self‐motivated behavior, can be used as a measure of spontaneous or stimulus‐evoked nociception in mice and rats.^50^ Burrowing behavior is displayed by all rodents and represents another very easy way to quantify the quality of life, as one simply needs to measure the weight of the burrowing material (eg gravel) at the beginning and end of any desired time period.^45^ This behavior has been shown to be disrupted by different visceral nociceptive processes such as laparotomy,^51^ nerve injury^52^ and colitis.^53^

### 4.3 Automated behavior analysis

Animal behaviors (feeding, drinking, grooming, licking, climbing‐resting‐climbing, anxiety and cognition related behaviors) are usually performed manually by well‐trained observers. Janyaro et al used a behavioral pain response scale (0‐4) to access the visceral pain triggered by traction on the ileocecal ligament in goats with ileitis.^7^ This manual approach is often labor intensive and time consuming, and can lack objectivity and reproducibility.^54^ Automated detection systems for assessing animal behaviors are worth considering in terms of saving time, achieving an objective analysis and reducing the need of well‐trained experimenters.^54^, ^55^ Hayashi et al^54^ used a software program (SCLABA, Noveltec Inc, Japan), originally developed for analyzing the scratching behaviors in rodents, to automate analysis of abdominal licking behavior associated with visceral pain in mice.

### 4.4 Telemetry

During animal experimentation, even when a person is simply present in a room, a mouse may hide the signs of pain, making monitoring of low to moderate pain very difficult.^56^ To overcome such problems, telemetry has been used, which allows monitoring without the presence of the investigator in the vicinity of the animal. Telemetry has been established to record physiological parameters such as heart rate, core body temperature, or blood pressure in mice.^57^ Nijsen et al^58^ used it as an appropriate tool for measuring the visceromotor and cardiovascular responses to averse, noxious duodenal distension continuously and simultaneously in the rats kept in a home cage, without additional handling‐related or restraint‐induced stress.

### 4.5 Electromyography

The visceromotor response (VMR) to colorectal distention (CRD), measured by electromyography (EMG), has been considered an objective indicator of VH.^59^, ^60^ It is performed to quantify the magnitude of the abdominal contractions evoked by CRD or other noxious stimulations. Several studies have utilized EMG to quantify the VH in different species of animal.^10^, ^11^, ^61^ For example, Ravnefjord et al^62^ studied the analgesic effect of pregabalin on visceral pain using EMG. Likewise, Yi et al^63^ performed EMG to investigate the maternal separation‐induced VH in colorectally distended rats.

### 4.6 Manometry

Manometry measures the pressures and the pattern of muscle contractions, particularly in the hollow visceral organs. Esophageal manometry is used to diagnose the conditions due to abnormalities in the contractions and strength of the esophageal muscle or in the sphincter at the lower end of the esophagus that results in pain, heartburn, and dysphagia. It is also used to study the visceral pain induced during CRD in irritable bowel syndrome (IBS) and the efficacy of different analgesics.^62^ The applicability of noninvasive, surgery‐free manometry of intracolonic pressure for assessing VMRs to CRD in mice has been reported.^64^ The effects of transcutaneous electrical nerve stimulation (TENS) on esophageal motility and pain sensitivity were assessed in graded intraesophageal balloon distended human patients using computerized esophageal manometry and it was reported that TENS attenuates noncardiac chest pain of esophageal origin.^65^

### 4.7 Pain biomarkers

Proposed biomarkers of pain include body weight decreases and autonomic nervous system manifestations such as pupil dilation, blood pressure, blood flow, respiration, heart rate, skin conductance, fecal corticosterone, plasma norepinephrine, and heart rate variability.^45^ Prostaglandin E2 (PGE2) is the predominant eicosanoid released after surgical trauma and has been associated with inflammation, pain, and fever, which result from the action of PGE2 on peripheral sensory neurons and on central sites within the spinal cord and brain.^66^ PGE2 increases thermal and mechanical hypersensitivity,^67^ exerting its actions by acting on a group of G‐protein‐coupled receptors (GPCRs). c‐Fos and pERK can be used as markers for neuronal activation following noxious stimulation and tissue injury.^68^ Kotani et al^69^ found significantly increased plasma concentrations of adrenal hormones (epinephrine, norepinephrine and cortisol) in patients after 1 hour and within a day of abdominal surgery.

### 4.8 Electrophysiology

The study of the electrical activity of nociceptors using exquisite electrophysiological techniques can offer an objective means of assessing visceral pain. Different studies have utilized electrophysiological approaches to investigate the mechanism of visceral pain. Referred hyperalgesia, an important characteristic of visceral pain, has also been investigated using electrophysiological experimentation. The electrical activity of ON‐like cells in the rostral ventromedial medulla has been assessed after intracolonic instillation of capsaicin.^70^ Reactive oxygen species (ROS) play a dual role in physiological and pathophysiological reactions, and have been used extensively in different inflammatory and neuropathic pain models.^71^, ^72^ Electrophysiological and behavioral studies have been performed to address the role of ROS in the central nucleus of amygdala in a visceral pain model induced by intracolonic zymosan.^73^

## 5 EXPERIMENTAL MODELS OF VISCERAL PAIN

Animal pain models that have been developed for pain research can be classified into acute, inflammatory, neuropathic, and clinically oriented pain models. Visceral pain models are used to study afferent nerve traffic during noxious stimulation at the level of the visceral organ. It is important to note that these models can be used for investigating the stimulus‐response patterns both during physiological (non‐painful) and pathological stimulation (inflammatory mediators) of the visceral organ. Previously, different models were developed to study the mechanism of visceral pain using irritants such as acetic acid, hypertonic saline, phenylquinone, among others, injected intraperitoneally but this method resulted in a nonselective action on the viscera and moreover produced a writhing behavior that was irreversible. The method also caused the animals suffering and, consequently, newer methods were invented to apply a finite noxious stimulation specifically to individual organs such as colon, urinary bladder, stomach, uterus and ovary. Recently, these models have fallen into disfavor and have been largely replaced with hollow organ balloon distension, which reproduces in humans the quality, location, and intensity of actual visceral pain. Christianson and Gebhart^74^ have studied colon sensitivity to luminal distension in mice. The distension of hollow organs produces several quantifiable responses including contraction of abdominal and pelvic muscles, and increased blood pressure and heart rates can be measured by electromyography and surgical implantation of an arterial catheter, respectively. In these studies, a balloon is inserted into the hollow organ and distended to provide a noxious stimulus to the wall of the organ.^75^ For ovarian pain, a new technique has been recently presented to determine the minimum alveolar concentration (MAC) in anesthetized dogs by applying a force to the ovary and ovarian ligament which can evoke purposeful movements. This model has been validated for induction of pelvic visceral pain.^6^

### 5.1 Inflammatory pain models

The objective of this model is to provoke a painful condition that mimics the natural inflammation induced by chronic visceral pain. Inflammatory pain is a big health concern causing suffering to millions in both humans and animals, especially during chronic inflammatory bowel diseases. The inflammatory pain model has helped scientists to understand the underlying mechanism of inflammatory pain and evaluate potential treatments. Table 2 summarizes the induction technique, and the purpose and important findings of inflammation‐induced visceral pain models. To induce inflammation, irritating substances (TNBS, zymosan, acetic acid, acrolin, cyclophosphamide etc) or the inflammatory mediator are directly injected into the esophagus, colon, ileum and urinary bladder of experimental animals. After the induction of inflammation, the pain responses are measured over a specified time (hours to days) by observing the withdrawal latency, EMG and analgesiometry.

**TABLE 2:** Inflammatory models for the study of visceral pain

Animal	Inflammatory agents	Injection site	Purpose of the study	Findings	References
Rat	TNBS in 50% ethanol	Colon	To develop a simple and reproducible model of chronic colonic inflammation by the intraluminal instillation of a solution containing a barrier breaker and a hapten	The combined administration of TNBS and ethanol resulted in the development of severe, transmural, granulomatous inflammation of the distal colon which may be useful for the study of the etiopathogenesis of chronic intestinal inflammation as well as providing an inexpensive model suitable for assessing potential treatments	Morris et al^87^
Rat	Dibutyltin dichloride, 10% ethanol	Tail vein injection, orally	Nociception in persistent pancreatitis and responsivity of Morphine	Animals with the dibutyltin dichloride‐induced experimental pancreatitis expressed serum, histologic, and behavioral characteristics similar in duration to those present during acute attacks experienced by patients with chronic pancreatitis and pain‐related measures were found to be abrogated by morphine	Vera‐Portocarrero et al^13^
Female rat	0.2% acetic acid	Intravesicular	To determine in female rats whether abdominal muscle discharges during normal voiding and to describe the effect of bladder irritation on this visceromotor activity	Acetic acid infusion reduced the inter contraction interval and increased bladder contraction duration. The afferents activating the visceromotor reflex during normal voiding and the increased reflex in response to acetic acid are probably both carried by the pelvic nerve. Abdominal muscle activity induced by bladder distension has been considered to be a pain marker	Cruz and Downie^77^
Mice	Zymosan	Colon	Examination of contributions of 2 proteins, TRPV1 and ASIC3, on development of behavioral hypersensitivity and assessment of the function of colon mechanoreceptors of hypersensitive mice	Zymosan sensitized the colonic mechanoreceptors acutely in vitro and chronic behavioral hypersensitivity (≥7 wk) in the quiescent inflammation. TRPV1 and ASIC3 proteins may be important peripheral mediators for development of functional visceral hypersensitivity	Jones et al^8^
Mice	Acrolein	Intravesicular	Evaluation of the severity of cystitis in response to increasing doses of acrolein through direct intravesicular administration	Intravesical instillation of acrolein produces dose‐dependent cystitis	Bjorling et al^78^
Guinea pig	TNBS	Ileal lumen	Morphological and functional changes in neurons projecting to the ileal mucosa at the early stage after inflammatory damage	Inflammation may be an important contributing factor to the neuronal hyperexcitability at the acute stage of inflammation	Nurgali et al^76^

Animals	Inflammatory agents	Injection site/stimuli	Purpose of the study	Findings	References
Wild mice	Intraluminal distension (100‐120 mmHg)	Colorectal	To identify the extrinsic nerve pathway(s) underlies nociception from the colorectum to the spinal cord of rodents	The visceral pain pathway activated by acute noxious distension of the terminal 15 mm of mouse colorectum is transmitted predominantly, if not solely, through rectal/pelvic afferent nerve fibers to the spinal cord. The sensory neurons of this spinal afferent pathway lie primarily in the lumbosacral region of the spinal cord, between L_6_ and S_1_	Kyloh et al^88^
Male rat	Balloon	Colon	Balloon distension of colon to investigate nonsteroidal anti‐inflammatory drug's effectiveness in CRD‐induced visceral pain model	Metamizole, dexketoprofen and meloxicam show antinociceptive effect with different duration of action on CRD‐induced visceral pain model	Baskın et al^89^
Rat	Intra‐colonic infusion of 0.5% acetic acid	Intra‐colonic	To investigate whether NO‐ mediated colonic motility was altered in rat IBS model, using different forms of NO‐synthase (NOS) inhibitors	The mean pressure values of spontaneous contractions and KCL (80 mmol/L) responses of distal colonic segments were similar in normal and IBS rats. L‐NAME and ARL‐17477 significantly increased the mean pressure of spontaneous colonic contractions in normal rats versus own base values (*P* < .05), but this increase did not significantly different when compared to IBS rats	Temiz et al^90^
Rat	TNBS	Ileum	Investigation of the visceral hypersensitivity provoked by TNBS‐induced ileitis rats	Transmural ileitis including granuloma and VH	Shah et al^10^
Rat	0.5% acetic acid	Intra‐colonic	To investigate in vitro effects of varenicline on spontaneous contractile responses of proximal and distal colon smooth muscle in control and IBS rats	Varenicline significantly decreased the mean pressure of spontaneous colonic contractions in both control and IBS rats compared to their baseline values in only distal colon (*P* < .05). The relaxation responses of distal colons were not significantly different between IBS and control groups (*P* > .05)	Kaya Temiz et al^91^
Goat	TNBS	Ileum	To testify the effect of electroacupuncture (EA) on ileitis‐provoked VH, and to confirm whether EA attenuates VH through JAK2/STAT3) signaling pathway in the PAG‐RVM‐SDH axis	EA attenuates VH probably through inhibiting JAK2/STAT3 signaling pathway in the PAG‐RVM‐SDH axis	Wan et al^7^

Abbreviations: ASIC3, Acid sensing ion channel‐3; CRD, Colorectal Distension; EMG, electromyography; IBD, inflammatory bowel disease; IBS, irritable bowel syndrome; IL‐1β, Interleukin‐1 beta; IL‐6, Interleukin‐6; JAK, Janus Kinase; NO, Nitric Oxide; PAG, periaqueductal gray; RVM, rostral ventromedial medulla; SDH, spinal dorsal horn; STAT, signal transducers and activators of transcription; TNBS, 2,4,6‐trinitrobenzene sulfonic acid; TNFα, Tumor Necrosis Factor‐alpha; TRPV1, Transient Receptor Potential Vanilloid Type 1; UBD, urinary bladder distention; VH, visceral hypersensitivity.

TNBS diluted in ethanol is used as a hapten to disrupt the mucosal barrier of hollow visceral organs.^75^ It has been regarded as a model for the study of ulcerative colitis and CD. TNBS/ethanol solution was injected into the ileal lumen of guinea pigs to elucidate the pathophysiology of ileal disease and the morphological and functional changes in neurons projecting to the ileal mucosa in the early stage after inflammatory damage.^76^ Like TNBS, zymosan, an insoluble carbohydrate from the yeast cell wall, has also been used to induce colitis for studying the association of short‐term sensitization of mechanoreceptors with long‐term hypersensitivity to colon distention in the mice.^8^ However, the severity of inflammation is milder compared to that caused by TNBS and generally does not produce mucosal ulceration.

An experimental model for the study of visceral pain associated with urinary bladder inflammation is developed by intravesicular injection of 0.2% acetic acid,^77^ zymosan^9^ or acrolein.^78^ Initially, the intraperitoneal injection of cyclophosphamide (an antitumor agent) was employed to produce bladder inflammation‐associated visceral pain,^79^ but although the cyclophosphamide produces selective cystitis via its metabolite, acrolein, in the bladder, it has a severe systemic toxic effect that can complicate the evaluation of bladder pain.

### 5.2 Traction pain models

Traction is the act of drawing or pulling the tissues or organ containing nociceptors from its normal position. It is important to note that the visceral organs are highly sensitive to traction, distension, ischemia and inflammation, which ultimately evoke visceral pain. Stretching or traction of the mesentery in the patients undergoing abdominal surgery causes a strong autonomic reaction. The induction technique, aims and key results of different traction models have been outlined chronologically in Table 3. Visceral pain may be induced by distension of the hollow viscera or of the capsule of parenchymal viscera and by peritoneal traction or stretching.^80^ Traction on mesentery as an experimental model of visceral nociception in anesthetized rats was developed for comparison of noxious mechanical and chemical stimulation of the mesentery.^81^ Boscan et al^6^ have established an ovarian ligament traction model and assessed the MAC in anesthetized dogs. Boscan et al^82^ also studied the anesthetic‐sparing effect of maropitant, a neurokinin‐1 receptor antagonist, during noxious visceral stimulation of the ovary and ovarian ligament in dogs. Likewise, Janyaro et al^7^ have established TNBS‐ileitis induced visceral pain triggered by traction on the ileocecal ligament. This model seems more effective for studying the mechanism of visceral pain because the TNBS injection into the ileum and ileocecal ligament traction potentiates the sensitization of thoracolumbar spinal afferent pathways of visceral pain.

**TABLE 3:** Traction models for the study of visceral pain

Animals	Traction on	Purpose of the study	Findings	References
Rat	Mesentery	Comparison of noxious mechanical and chemical stimulation of the mesentery	The measurement of the cardiovascular and gastric responses of anaesthetized rats to traction on the mesentery is a suitable method to investigate acute visceral nociception	Holzer‐Petsche and Brodacz^81^
Dog	Ovary, ovarian ligament	Study of visceral pain by stimulating the ovarian ligament	The ovarian stimulation model is an adequate and repeatable means of producing visceral stimulation to determine MAC	Boscan et al^6^
Dog	Ovary, ovarian ligament	Determination of the anesthetic‐sparing effect of maropitant, a neurokinin‐1 receptor antagonist, during noxious visceral stimulation of the ovary and ovarian ligament in dogs	Decrease in the sevoflurane MAC requirement during ovary and ovarian ligament stimulation after intravenous administration of maropitant	Boscan et al^82^
Cat	Ovary, ovarian ligament	Investigation of antinociceptive effects of maropitant and to determine the MAC sparing effect of this drug in cats during visceral noxious stimulus	Intravenous administration of maropitant reduced the sevoflurane requirements	Niyom^20^
Goat	Ileocecal ligament with TNBS induced ileitis	Quantification of the intensity of visceral pain using ileocecal ligament traction on an inflamed ileum in goats	The traction force correlated positively with pain‐behavior responses and provoked an apparent, stable, and reproducible ileum‐derived pain	Janyaro et al^7^

Abbreviations: MAC, minimum alveolar concentration; NK‐1, neurokinin‐1.

### 5.3 Stress/genetic models of visceral pain

Various animal models, including early life stress, maternal separation stress, water avoidance stress, post‐traumatic stress, wrap restraint stress and adult stress models, have been developed as psychosocial stressors in an attempt to understand the mechanisms responsible for chronic visceral pain disorders in rodents that mimic IBS. Chronic visceral pain, an interaction of gene and environment, results from maladaptive changes in neuronal circuitry leading to neuroplasticity and aberrant neuronal activity‐induced signaling.^15^ Several other factors such as prenatal (sex, genetic and epigenetic), early life (neonatal maternal separation, early trauma) and adult (psychosocial stressors, anxiety, depression, stress hyper‐responsiveness, physical stress, intestinal infection and other visceral diseases or disorders) factors are responsible for not only exacerbating visceral sensitivity but may also be predisposing factors for the development of VH in later life.

The persistent effects of stress on visceral sensitivity could be attributed to epigenetic modulation of gene expression. A few researchers^17^, ^18^ have highlighted the epigenetically mediated mechanisms involved in stress‐induced visceral sensitivity, changes in DNA‐methylation and histone‐acetylation patterns within the brain and SDH of rats which have been linked to alterations in nociceptive signaling via increased expression of pro‐ and anti‐nociceptive gene expressions.^83^ Zheng et al^16^ compared somatosensory and visceral hyperalgesia with respect to the differential responses of peripheral pain regulatory pathways in a rat model of chronic, intermittent stress and reported that chronic stress induces both somatosensory and visceral hyperalgesia due to differential changes in endovanilloid and endocannabinoid pathways, and sodium channels in dorsal root ganglions innervating the lower extremities and pelvic viscera. Asano et al^84^ reported that oral administration of aminophylline supressed the VH provoked in maternally separated rats, acetic acid‐induced colitis rats and wrap restraint stress rats. The stressors/gene knockout, purpose of study and key results of different stress and genetic models for studying the underlying mechanism of visceral pain have been summarized chronologically in Table 4. Knockout models provide the opportunity to investigate the role of a specific gene in the regulation of colonic visceral sensitivity. Over the years, several studies have employed knockout models to unravel the gene‐specific underlying mechanism of VH. Earlier studies^84^, ^85^ reported that knockout of corticotropin‐releasing factor‐1 receptor attenuates the VH in response to different CRD pressures in mice. It is well known that the voltage‐gated sodium channel subtype Na_V_1.7 is a prerequisite for sensing acute and inflammatory somatic pain in mice and humans. However, its involvement in pain originating from the viscera is still poorly understood. Hockely et al^86^ investigated the role of Na_V_1.7 in visceral pain processing and the development of referred hyperalgesia using a conditional nociceptor‐specific Na_V_1.7 knockout mouse and reported that Na_V_1.7 knockout did not lead to any differences in pain‐related responses and referred hyperalgesia to noxious mechanical and chemical stimuli in nerve‐gut preparations in mouse.

**TABLE 4:** Stress/genetic models for the study of visceral pain

Animals	Stressor/ Knockout gene	Purpose of the study	Findings	References
Adult male rat	ICV cannulated rats were exposed to repeated water avoidance stress and administered with TSA, a potent histone deacetylase inhibitor	To study the importance of epigenetic mechanisms in visceral pain induced by chronic water avoidance stress	Stressed rats exhibited visceral hypersensitivity that was significantly attenuated by TSA. Compared to SHAM controls, methylation of the GR gene was increased following WAS while expression of the GR gene was decreased. Methylation of the CRF promoter was decreased with WAS with a concomitant increase in CRF expression	Tran et al^18^
Rat	WRS Model	To test the effect of a bronchodilator, aminophylline on stress‐induced defecation and visceral hypersensitivity and defecation in irritable bowel syndrome	Aminophylline suppressed maternal separation‐ and acetic acid administration‐induced visceral hypersensitivity to CRD, which was mediated by both A2AARs and A2BARs	Asano et al^84^
Adult mice	Knockout model	To evaluate the effect of Tetrodotoxin on pure visceral pain induced by intracolonic instillation of capsaicin and mustard oil and its effect in a Na_v_1.7 Knockout Mice	No differences in pain‐related responses and referred hyperalgesia with respect to their control mice littermates when they were instilled intra‐colonically with capsaicin and mustard oil or treated intraperitoneally with cyclophosphamide	Gonzalez‐Cano et al^92^
Male and female rat	Knockout model	To examine the role of NaV1.7 in visceral pain processing and the development of referred hyperalgesia using a conditional nociceptor‐specific NaV1.7 knockout mouse (NaV1.7Nav1.8) and selective small‐molecule NaV1.7 antagonist PF‐5198007	NaV1.7 (in NaV1.8‐expressing neurons) contributes to defined pain pathways in a modality‐dependent manner, modulating somatic noxious heat pain, but is not required for visceral pain processing, and advocate that pharmacological block of NaV1.7 alone in the viscera may be insufficient in targeting chronic visceral pain	Hockley et al^86^

Abbreviations: CRD, Colorectal Distension; CRF, Corticotropin Releasing Factor; ICV, Intracerebroventricular; TSA, Trichostatin‐A; WAS, water avoidance stress; WRS, wrap restraint stress.

## 6 CONCLUSIONS

Visceral pain, an unpleasant sensory and emotional experience, results from the activation of nociceptors. Damaged tissue or inflammatory cells release different mediators such as histamine, 5‐HT, BK, PG, IL, and NGF that activate or modify the stimulus response properties of C‐ and Aδ‐fibers of thoracic, abdominal or pelvic organs via specific receptors or ion channels. Sensitization of the peripheral afferents, spinal, or brainstem/thalamic neurons promotes chronic visceral pain signaling. Although precise measurement of visceral pain is very difficult, experimenters have developed different methods to estimate its severity. Among them, EMG, electrophysiology, telemetry, and grimace scales are the most frequently used tools due to the advantages of labor and time savings, and good objectivity and reproducibility. Different animal models have provided multiple lines of pivotal evidence to improve our understanding of the underlying pathophysiological mechanism of visceral pain. Because of the complex and multifactorial nature of visceral pain, involving higher cognitive and cortical function, as well as complicated interactions between biological, psychological, and sociological variables, no animal model is able to mimic visceral pain completely. Inflammatory and traction models are thought to be very insightful for studying the underlying mechanism of visceral pain and hypersensitivity since they mimic the natural disease condition and post‐operative pain in humans and animals. Traction produces an adequate and repeatable visceral stimulation that results in a strong autonomic reaction and seems appropriate for studying the efficacies of novel analgesics and anesthetic agents. The inflammatory‐traction model can be used for the intervention studies of visceral pain because the local inflammatory mediators and stretching of the mesentery and ligaments provide a more intense stimulation of nociceptors.

## CONFLICT OF INTEREST

None.

## AUTHOR CONTRIBUTIONS

MKS contributed to conception and outline of the manuscript. BR drafted the manuscript. MKS and BR revised the manuscript. Both authors read and approved the final manuscript.

## References

[1] Davis MP . Drug management of visceral pain: concepts from basic research. Pain Res Treat. 2012;2012:265605. 22619712 10.1155/2012/265605 PMC3348642

[2] Greenwood‐Van Meerveld B , Prusator DK , Johnson AC . Animal models of gastrointestinal and liver diseases. Animal models of visceral pain: pathophysiology, translational relevance, and challenges. Am J Physiol Gastrointest Liver Physiol. 2015;308(11):G885‐G903. 25767262 10.1152/ajpgi.00463.2014

[3] Zhang F , Wu L , Zhao J , et al. Neurobiological mechanism of acupuncture for relieving visceral pain of gastrointestinal origin. Gastroenterol Res Pract. 2017;2017:5687496. 28243252 10.1155/2017/5687496 PMC5294365

[4] Lehne RA . Pharmacology for Nursing Care. 8th ed. St. Louis, MO: Elsevier Health Sciences; 2013. Accessed May 5, 2020.

[5] Sengupta JN . Visceral pain: the neurophysiological mechanism. Handb Exp Pharmacol. 2009;194:31‐74. 10.1007/978-3-540-79090-7_2 PMC3156094 19655104

[6] Boscan P , Monnet E , Mama K , et al. A dog model to study ovary, ovarian ligament and visceral pain. Vet Anaesth Analg. 2011;38(3):260‐266. 21492392 10.1111/j.1467-2995.2011.00611.x

[7] Janyaro H , Wan J , Tahir AH , Shah MK , Li XJ , Ding MX . Visceral pain triggered by traction on the ileocecal ligament with ileitis. J Pain Res. 2016;9:745‐755. 27757049 10.2147/JPR.S115127 PMC5053401

[8] Jones RCW , Otsuka E , Wagstrom E , Jensen CS , Price MP , Gebhart GF . Short‐term sensitization of colon mechanoreceptors is associated with long‐term hypersensitivity to colon distention in the mouse. Gastroenterology. 2007;133(1):184‐194. 17553498 10.1053/j.gastro.2007.04.042

[9] DeBerry J , Ness TJ , Robbins MT , Randich A . Inflammation‐induced enhancement of the visceromotor reflex to urinary bladder distention: modulation by endogenous opioids and the effects of early‐in‐life experience with bladder inflammation. J Pain. 2007;8(12):914‐923. 17704007 10.1016/j.jpain.2007.06.011 PMC4012257

[10] Shah MK , Wan J , Janyaro H , Tahir AH , Cui L , Ding MX . Visceral hypersensitivity is provoked by 2,4,6‐trinitrobenzene sulfonic acid‐induced ileitis in rats. Front Pharmacol. 2016;7:214. 27499743 10.3389/fphar.2016.00214 PMC4956665

[11] Wan J , Ding Y , Tahir AH , et al. Electroacupuncture attenuates visceral hypersensitivity by inhibiting JAK2/STAT3 signaling pathway in the descending pain modulation system. Front Neurosci. 2017;11:644. 29209161 10.3389/fnins.2017.00644 PMC5701938

[12] Shah MK , Ding Y , Wan J , et al. Electroacupuncture intervention of visceral hypersensitivity is involved in PAR‐2‐activation and CGRP‐release in the spinal cord. Sci Rep. 2020;10(1):11188. 32636402 10.1038/s41598-020-67702-2 PMC7341736

[13] Vera‐Portocarrero LP , Lu Y , Westlund KN . Nociception in persistent pancreatitis in rats: effects of morphine and neuropeptide alterations. Anesthesiology. 2003;98(2):474‐484. 12552208 10.1097/00000542-200302000-00029 PMC4654116

[14] Lightman SL . The neuroendocrinology of stress: a never ending story. J Neuroendocrinol. 2008;20(6):880‐884. 18601712 10.1111/j.1365-2826.2008.01711.x

[15] Greenwood‐Van Meerveld B , Johnson AC . Stress‐induced chronic visceral pain of gastrointestinal origin. Front Syst Neurosci. 2017;11:86. 29213232 10.3389/fnsys.2017.00086 PMC5702626

[16] Zheng G , Hong S , Hayes JM , Wiley JW . Chronic stress and peripheral pain: evidence for distinct, region‐specific changes in visceral and somatosensory pain regulatory pathways. Exp Neurol. 2015;273:301‐311. 26408049 10.1016/j.expneurol.2015.09.013 PMC4644500

[17] Hong S , Zheng G , Wiley JW . Epigenetic regulation of genes that modulate chronic stress‐induced visceral pain in the peripheral nervous system. Gastroenterology. 2015;148(1):148‐157.e7. 25263804 10.1053/j.gastro.2014.09.032 PMC4274248

[18] Tran L , Chaloner A , Sawalha AH , Greenwood V‐M . Importance of epigenetic mechanisms in visceral pain induced by chronic water avoidance stress. Psychoneuroendocrinology. 2013;38(6):898‐906. 23084728 10.1016/j.psyneuen.2012.09.016

[19] Duda D , Lorenz W , Celik I . Mesenteric traction syndrome during the operation of aneurysms of the abdominal aorta–histamine release and prophylaxis with antihistaminics. Anaesthesiol Reanim. 2003;28(4):97‐103. 14528656

[20] Niyom S .Evaluation of potential analgesic drugs using new models to study pain in dogs and cats. 2013 https://dspace.library.colostate.edu/handle/10217/80168 Accessed June 1, 2017

[21] Sherman R . Abdominal Pain. Ann Intern Med. 1980;92(3):452.

[22] Kruszka PS , Kruszka SJ . Evaluation of acute pelvic pain in women. Am Fam Physician. 2010;82(2):141‐147. 20642266

[23] Heymen S , Maixner W , Whitehead WE , Klatzkin RR , Mechlin B , Light KC . Central processing of noxious somatic stimuli in patients with irritable bowel syndrome compared with healthy controls. Clin J Pain. 2010;26(2):104‐109. 20090435 10.1097/AJP.0b013e3181bff800 PMC4321817

[24] Williams RE , Hartmann KE , Sandler RS , Miller WC , Steege JF . Prevalence and characteristics of irritable bowel syndrome among women with chronic pelvic pain. Obstet Gynecol. 2004;104(3):452‐458. 15339753 10.1097/01.AOG.0000135275.63494.3d

[25] Bouhassira D , Moisset X , Jouet P , Duboc H , Coffin B , Sabate JM . Changes in the modulation of spinal pain processing are related to severity in irritable bowel syndrome. Neurogastroenterol Motil. 2013;25(7):623‐e468. 10.1111/nmo.12123 23551988

[26] Sinatra R . Role of COX‐2 inhibitors in the evolution of acute pain management. J Pain Symptom Manage. 2002;24(1 Suppl):S18‐S27. 12204484 10.1016/s0885-3924(02)00410-4

[27] Delmas P , Korogod SM , Coste B . Noxious mechanosensation In: Wood JN, ed. The Oxford Handbook of the Neurobiology of Pain. Oxford, England: Oxford University Press; 2018.

[28] D’Mello R , Dickenson AH . Spinal cord mechanisms of pain. Br J Anaesth. 2008;101(1):8‐16. 18417503 10.1093/bja/aen088

[29] Jay GW . Chronic Pain, 1st ed. North Carolina, USA: CRC Press; 2007.

[30] Cervero F , Laird JMA . Visceral pain. Lancet. 1999;353(9170):2145‐2148. 10382712 10.1016/S0140-6736(99)01306-9

[31] Cervero F . Pathophysiology of visceral pain. Rev Dor. 2014;15(2):133‐138.

[32] Scholz J , Woolf CJ . Can we conquer pain? Nat Neurosci. 2002;5(11s):1062‐1067. 12403987 10.1038/nn942

[33] Ganapathy MK , Reddy V , Tadi P . Neuroanatomy, Spinal Cord Morphology. Treasure Island: StatPearls Publishing; 2019 http://www.ncbi.nlm.nih.gov/pubmed/31424790. Accessed May 6, 2020. 31424790

[34] Cohen MJ , Jangro WC , Neff D . Pathophysiology of pain In: Challenging Neuropathic Pain Syndromes: Evaluation and Evidence‐Based Treatment. Amsterdam, Netherlands: Elsevier Inc.; 2018:1‐5.

[35] Ramesh G , Maclean AG , Philipp MT . Cytokines and chemokines at the crossroads of neuroinflammation, neurodegeneration, and neuropathic pain. Mediators Inflamm. 2013;2013:480739. 23997430 10.1155/2013/480739 PMC3753746

[36] Devi LA . G‐protein‐coupled receptor dimers in the lime light. Trends Pharmacol Sci. 2000;21(9):324‐326. 10973076 10.1016/s0165-6147(00)01519-4

[37] Watkins LR , Hutchinson MR , Ledeboer A , Wieseler‐Frank J , Milligan ED , Maier SF . Glia as the “bad guys”: Implications for improving clinical pain control and the clinical utility of opioids. Brain Behav Immun. 2007;21(2):131‐146. 17175134 10.1016/j.bbi.2006.10.011 PMC1857294

[38] Zieglgänsberger W . Substance P and pain chronicity. Cell Tissue Res. 2019;375(1):227‐241. 30284083 10.1007/s00441-018-2922-y PMC6335504

[39] Braz J , Solorzano C , Wang X , Basbaum AI . Transmitting pain and itch messages: a contemporary view of the spinal cord circuits that generate gate control. Neuron. 2014;82(3):522‐536. 24811377 10.1016/j.neuron.2014.01.018 PMC4492533

[40] Khurana I . Essentials of Medical Physiology. Delhi, India: Elsevier; 2008.

[41] Graham DM , Hampshire V . Methods for measuring pain in laboratory animals. Lab Anim (NY). 2016;45(3):99‐101. 26886673 10.1038/laban.962

[42] Laux‐Biehlmann A , Boyken J , Dahllöf H , Schmidt N , Zollner TM , Nagel J . Dynamic weight bearing as a non‐reflexive method for the measurement of abdominal pain in mice. Eur J Pain (United Kingdom). 2016;20(5):742‐752. 10.1002/ejp.800 26684879

[43] Griffioen MA , Dernetz VH , Yang GS , Griffith KA , Dorsey SG , Renn CL . Evaluation of dynamic weight bearing for measuring nonevoked inflammatory hyperalgesia in mice. Nurs Res. 2015;64(2):81‐87. 25738619 10.1097/NNR.0000000000000082 PMC4351786

[44] Quadros AU , Pinto LG , Fonseca MM , Kusuda R , Cunha FQ , Cunha TM . Dynamic weight bearing is an efficient and predictable method for evaluation of arthritic nociception and its pathophysiological mechanisms in mice. Sci Rep. 2015;5:14648. 26511791 10.1038/srep14648 PMC4625149

[45] Mogil JS . The measurement of pain in the laboratory rodent In: The Oxford Handbook of the Neurobiology of Pain. Oxford, England: Oxford University Press; 2019.

[46] Sotocinal SG , Sorge RE , Zaloum A , et al. The Rat Grimace Scale: a partially automated method for quantifying pain in the laboratory rat via facial expressions. Mol Pain. 2011;7:55. 21801409 10.1186/1744-8069-7-55 PMC3163602

[47] Langford DJ , Bailey AL , Chanda ML , et al. Coding of facial expressions of pain in the laboratory mouse. Nat Methods. 2010;7(6):447‐449. 20453868 10.1038/nmeth.1455

[48] Evangelista MC , Watanabe R , Leung VSY , et al. Facial expressions of pain in cats: the development and validation of a Feline Grimace Scale. Sci Rep. 2019;9(1):19128. 31836868 10.1038/s41598-019-55693-8 PMC6911058

[49] Leung VSY , Benoit‐Biancamano MO , Pang DSJ . Performance of behavioral assays: The Rat Grimace Scale, burrowing activity and a composite behavior score to identify visceral pain in an acute and chronic colitis model. Pain Reports. 2019;4(2):e718. 10.1097/PR9.0000000000000712 PMC6455688 31041420

[50] Deuis JR , Dvorakova LS , Vetter I . Methods used to evaluate pain behaviors in rodents. Front Mol Neurosci. 2017;10:284. 28932184 10.3389/fnmol.2017.00284 PMC5592204

[51] Jirkof P , Cesarovic N , Rettich A , Nicholls F , Seifert B , Arras M . Burrowing behavior as an indicator of post‐laparotomy pain in mice. Front Behav Neurosci. 2010;4:165. 21031028 10.3389/fnbeh.2010.00165 PMC2965018

[52] Andrews N , Legg E , Lisak D , et al. Spontaneous burrowing behaviour in the rat is reduced by peripheral nerve injury or inflammation associated pain. Eur J Pain. 2012;16(4):485‐495. 22396078 10.1016/j.ejpain.2011.07.012

[53] Jirkof P , Leucht K , Cesarovic N , et al. Burrowing is a sensitive behavioural assay for monitoring general wellbeing during dextran sulfate sodium colitis in laboratory mice. Lab Anim. 2013;47(4):274‐283. 23828853 10.1177/0023677213493409

[54] Hayashi E , Kobayashi T , Shiroshita Y , Kuratani K , Kinoshita M , Hara H . An automated evaluation system for analyzing antinociceptive effects on intracolonic capsaicin‐induced visceral pain‐related licking behavior in mice. J Pharmacol Toxicol Methods. 2011;64(2):119‐123. 21440074 10.1016/j.vascn.2011.03.004

[55] Bello‐Arroyo E , Roque H , Marcos A , et al. MouBeAT: a new and open toolbox for guided analysis of behavioral tests in mice. Front Behav Neurosci. 2018;12:201. 30245618 10.3389/fnbeh.2018.00201 PMC6137138

[56] Van Sluyters RC , Obernier JA . Guidelines for the care and use of mammals in neuroscience and behavioral research. Contemp Top Lab Anim Sci. 2004;43(2):48‐52.

[57] Arras M , Rettich A , Cinelli P , Kasermann HP , Burki K . Assessment of post‐laparotomy pain in laboratory mice by telemetric recording of heart rate and heart rate variability. BMC Vet Res. 2007;3(1):16. 17683523 10.1186/1746-6148-3-16 PMC1965463

[58] Nijsen MJMA , Ongenae NGH , Coulie B , Meulemans AL . Telemetric animal model to evaluate visceral pain in the freely moving rat. Pain. 2003;105(1‐2):115‐123. 14499427 10.1016/s0304-3959(03)00170-2

[59] Deiteren A , Vermeulen W , Moreels TG , Pelckmans PA , De Man JG , De Winter BY . The effect of chemically induced colitis, psychological stress and their combination on visceral pain in female Wistar rats. Stress. 2014;17(5):431‐444. 25089934 10.3109/10253890.2014.951034

[60] Vermeulen W , Joris GDM , Heiko UDS , et al. Role of TRPV1 and TRPA1 in visceral hypersensitivity to colorectal distension during experimental colitis in rats. Eur J Pharmacol. 2013;698(1‐3):404‐412. 23099257 10.1016/j.ejphar.2012.10.014

[61] Tahir AH , Wan J , Shah MK , Janyaro H , Li XJ , Ding MX . A novel model for studying ileitis‐induced visceral hypersensitivity in goats. Acta Vet Scand. 2016;58(1):72. 27716368 10.1186/s13028-016-0253-0 PMC5052972

[62] Ravnefjord A , Brusberg M , Larsson H , Lindström E , Martínez V . Effects of pregabalin on visceral pain responses and colonic compliance in rats. Br J Pharmacol. 2008;155(3):407‐416. 18574457 10.1038/bjp.2008.259 PMC2567880

[63] Yi L , Zhang H , Sun H , et al. Maternal separation induced visceral hypersensitivity from childhood to adulthood. J Neurogastroenterol Motil. 2017;23(2):306‐315. 28238254 10.5056/jnm16089 PMC5383126

[64] Arvidsson S , Larsson M , Larsson H , Lindström E , Martinez V . Assessment of visceral pain‐related pseudo‐affective responses to colorectal distension in mice by intracolonic manometric recordings. J Pain. 2006;7(2):108‐118. 16459276 10.1016/j.jpain.2005.09.003

[65] Börjesson M , Pilhall M , Eliasson T , Norssell H , Mannheimer C , Rolny P . Esophageal visceral pain sensitivity: Effects of TENS and correlation with manometric findings. Dig Dis Sci. 1998;43(8):1621‐1628. 9724141 10.1023/a:1018886309364

[66] Funk CD . Prostaglandins and leukotrienes: advances in eicosanoid biology. Science. 2001;294(5548):1871‐1875. 11729303 10.1126/science.294.5548.1871

[67] Domenichiello AF , Wilhite BC , Keyes GS , Ramsden CE . A dose response study of the effect of prostaglandin E2 on thermal nociceptive sensitivity. Prostaglandins Leukot Essent Fat Acids. 2017;126:20‐24. 10.1016/j.plefa.2017.08.015 PMC5679719 29031391

[68] Bullitt E . Expression of C‐fos‐like protein as a marker for neuronal activity following noxious stimulation in the rat. J Comp Neurol. 1990;296(4):517‐530. 2113539 10.1002/cne.902960402

[69] Kotani N , Hashimoto H , Sato Y , et al. Preoperative intradermal acupuncture reduces postoperative pain, nausea and vomiting, analgesic requirement, and sympathoadrenal responses. Anesthesiology. 2001;95(2):349‐356. 11506105 10.1097/00000542-200108000-00015

[70] Sanoja R , Tortorici V , Fernandez C , Price TJ , Cervero F . Role of RVM neurons in capsaicin‐evoked visceral nociception and referred hyperalgesia. Eur J Pain. 2010;14(2):120.e1‐120.e9. 19443247 10.1016/j.ejpain.2009.04.006 PMC2914578

[71] Vaculin Simon S , Franek M , Vejrazka M . Role of oxidative stress in animal model of visceral pain. Neurosci Lett. 2010;477(2):82‐85. 20417688 10.1016/j.neulet.2010.04.037

[72] Bittar A , Jun J , La JH , Wang J , Leem JW , Chung JM . Reactive oxygen species affect spinal cell type‐specific synaptic plasticity in a model of neuropathic pain. Pain. 2017;158(11):2137‐2146. 28708760 10.1097/j.pain.0000000000001014 PMC5963270

[73] Ji G , Li Z , Neugebauer V . Reactive oxygen species mediate visceral pain‐related amygdala plasticity and behaviors. Pain. 2015;156(5):825‐836. 25734993 10.1097/j.pain.0000000000000120 PMC4402250

[74] Christianson JA , Gebhart GF . Assessment of colon sensitivity by luminal distension in mice. Nat Protoc. 2007;2(10):2624‐2631. 17948005 10.1038/nprot.2007.392

[75] Ness TJ , Lewis‐Sides A , Castroman P . Characterization of pressor and visceromotor reflex responses to bladder distention in rats: sources of variability and effect of analgesics. J Urol. 2001;165(3):968‐974. 11176524

[76] Nurgali K , Qu Z , Hunne B , Thacker M , Pontell L , Furness JB . Morphological and functional changes in guinea‐pig neurons projecting to the ileal mucosa at early stages after inflammatory damage. J Physiol. 2011;589(Pt 2):325‐339. 21098001 10.1113/jphysiol.2010.197707 PMC3043536

[77] Cruz Y , Downie JW . Abdominal muscle activity during voiding in female rats with normal or irritated bladder. Am J Physiol Regul Integr Comp Physiol. 2006;290(5):R1436‐R1445. 16373437 10.1152/ajpregu.00556.2005

[78] Bjorling DE , Elkahwaji JE , Bushman W , et al. Acute acrolein‐induced cystitis in mice. BJU Int. 2007;99(6):1523‐1529. 17346276 10.1111/j.1464-410X.2007.06773.x

[79] Boucher M , Meen M , Codron JP , Coudore F , Kemeny JL , Eschalier A . Cyclophosphamide‐induced cystitis in freely‐moving conscious rats: behavioral approach to a new model of visceral pain. J Urol. 2000;164(1):203‐208. 10840460

[80] Zakka TM , Teixeira MJ , Yeng LT . Abdominal visceral pain: clinical aspects. Rev Dor. 2013;14(4):311‐314.

[81] Holzer‐Petsche U , Brodacz B . Traction on the mesentery as a model of visceral nociception. Pain. 1999;80(1‐2):319‐328. 10204745 10.1016/s0304-3959(98)00233-4

[82] Boscan P , Monnet E , Mama K , Twedt DC , Congdon J , Steffey EP . Effect of maropitant, a neurokinin 1 receptor antagonist, on anesthetic requirements during noxious visceral stimulation of the ovary in dogs. Am J Vet Res. 2011;72(12):1576‐1579. 22126683 10.2460/ajvr.72.12.1576

[83] Meerveld BG , Johnson AC . Mechanisms of stress‐induced visceral pain. J Neurogastroenterol Motil. 2018;24(1):7‐18. 29291604 10.5056/jnm17137 PMC5753899

[84] Asano T , Tanaka KI , Tada A , et al. Aminophylline suppresses stress‐induced visceral hypersensitivity and defecation in irritable bowel syndrome. Sci Rep. 2017;7:40214. 28054654 10.1038/srep40214 PMC5214462

[85] Trimble N , Johnson AC , Foster A , Greenwood‐Van MB . Corticotropin‐releasing factor receptor 1‐deficient mice show decreased anxiety and colonic sensitivity. Neurogastroenterol Motil. 2007;19(9):754‐760. 17539891 10.1111/j.1365-2982.2007.00951.x

[86] Hockley JRF , González‐Cano R , Mcmurray S , et al. Visceral and somatic pain modalities reveal Na V 1.7‐independent visceral nociceptive pathways. J Physiol. 2017;5958(5958):2661‐2679. 10.1113/JP272837 PMC5390874 28105664

[87] Morris GP , Beck PL , Herridge MS , Depew WT , Szewczuk MR , Wallace JL . Hapten‐induced model of chronic inflammation and ulceration in the rat. Gastroenterology. 1989;96(3):795‐803. 2914642

[88] Kyloh M , Nicholas S , Zagorodnyuk VP , Brookes SJ , Spencer NJ . Identification of the visceral pain pathway activated by noxious colorectal distension in mice. Front Neurosci. 2011;5:16. 21390285 10.3389/fnins.2011.00016 PMC3046361

[89] Baskın V , Bilge SS , Bozkurt A , et al. Effect of nonsteroidal anti‐inflammatory drugs on colorectal distension‐induced visceral pain. Indian J Pharmacol. 2016;48(2):150‐154. 27114637 10.4103/0253-7613.178830 PMC4825431

[90] Temiz TK , Demir O , Simsek F , et al. Effect of nitrergic system on colonic motility in a rat model of irritable bowel syndrome. Indian J Pharmacol. 2016;48(4):424‐429. 27756955 10.4103/0253-7613.186189 PMC4980932

[91] Temiz TK , Demir O , Keskin‐Arslan E , Acar S , Karadas B , Koyluoglu G . Effect of varenicline on colonic motility in a rat model of experimental irritable bowel. Biomed Res. 2017;28(6):2743‐2748.

[92] González‐Cano R , Tejada MÁ , Artacho‐Cordón A , et al. Effects of tetrodotoxin in mouse models of visceral pain. Mar Drugs. 2017;15(6):188. 10.3390/md15060188 PMC5484138 28635651

